# Global distribution data for cattle, buffaloes, horses, sheep, goats, pigs, chickens and ducks in 2010

**DOI:** 10.1038/sdata.2018.227

**Published:** 2018-10-30

**Authors:** Marius Gilbert, Gaëlle Nicolas, Giusepina Cinardi, Thomas P. Van Boeckel, Sophie O. Vanwambeke, G. R. William Wint, Timothy P. Robinson

**Affiliations:** 1 Spatial Epidemiology Lab (SpELL), Université Libre de Bruxelles, Brussels, Belgium; 2 Fonds National de la Recherche Scientifique (FNRS), Brussels, Belgium; 3 Animal Production and Health Division (AGA), Food and Agriculture Organization of the United Nations, Rome, Italy; 4 Institute of Integrative Biology, ETH Zurich, Zurich, Switzerland; 5 Center for Diseases Dynamics Economics and Policy, Washington DC, USA; 6 Earth and Life Institute, Université catholique de Louvain, Louvain-la-Neuve, Belgium; 7 Environment Research Group Oxford (ERGO), Department of Zoology, Oxford, United Kingdom

**Keywords:** Agroecology, Biogeography, Risk factors, Environmental chemistry

## Abstract

Global data sets on the geographic distribution of livestock are essential for diverse applications in agricultural socio-economics, food security, environmental impact assessment and epidemiology. We present a new version of the Gridded Livestock of the World (GLW 3) database, reflecting the most recently compiled and harmonized subnational livestock distribution data for 2010. GLW 3 provides global population densities of cattle, buffaloes, horses, sheep, goats, pigs, chickens and ducks in each land pixel at a spatial resolution of 0.083333 decimal degrees (approximately 10 km at the equator). They are accompanied by detailed metadata on the year, spatial resolution and source of the input census data. Two versions of each species distribution are produced. In the first version, livestock numbers are disaggregated within census polygons according to weights established by statistical models using high resolution spatial covariates (dasymetric weighting). In the second version, animal numbers are distributed homogeneously with equal densities within their census polygons (areal weighting) to provide spatial data layers free of any assumptions linking them to other spatial variables.

## Background & Summary

Livestock play a key role in global food systems as the main source of animal protein (milk, meat and eggs), contribute to crop productivity through the provision of draught power and manure, and to the livelihoods and nutrition of poor households in low- and middle-income countries^1^ (LMICs). Livestock farming has a major impact on the environment, through greenhouse gas (GHG) emissions from enteric fermentation and manure, disruption of nitrogen and phosphorous cycles and indirect impacts on biodiversity and other ecosystem services through overgrazing and land-use change^2^. Livestock farming also bears public health implications through its role in food-borne disease transmission, the emergence and spread of infectious zoonotic diseases^3^ such as avian influenza^4^, Q-fever and MERS and its contribution to the global burden of antimicrobial resistance, linked to the routine abuse of those drugs in livestock production.^5,6^ Detailed, contemporary data sets on the global distribution of the most important species of farmed animals have a wide range of applications in understanding the social, economic, environmental, epidemiological and public health impacts of the livestock sector.

The gridded livestock of the world database (GLW 1) produced in 2007 had three objectives^7^: i) to collect, harmonize and disseminate subnational global livestock data, ii) to predict livestock numbers in areas with missing census counts (gap-filling), and iii) to provide a statistically-informed estimate of how livestock may be distributed within census units (downscaling). GLW 1 was produced at a spatial resolution of 0.0416666 decimal degrees (approximately 5 km at the equator). In 2014, an updated GLW 2 was published, benefiting from the availability of finer-scale and more contemporary input census data, from the improvement of the processing and from higher spatial resolution predictor variables that were used for downscaling^8^.

In this paper, we describe a new global, subnational livestock dataset (GLW 3) generated using Random Forests (RF), a machine-learning technique recently shown to provided more accurate gap-filling and disaggregation of livestock data than did the previously-used multivariate regression methods^9^. In addition to that important change in methodology, GLW 3 differs from the previous ones in three ways.

For each species, we now provide a detailed report that includes comprehensive metadata on the input census data for each country (e.g. year, resolution and source) and goodness-of-fit metrics of the models by continent and by the size of the administrative unit from which the census data came. This enables users to assess the quality of the estimates for each combination of species, country and size of census unit.

All species distributions are now available in two representations, termed dasymetric (DA) and areal-weighted (AW). The DA models correspond to previous GLW versions, whereby different animal densities are assigned to different pixels within a given census polygon according to the RF models. In contrast, the AW models simply spread individuals of a census polygon evenly, and the density of animals in each pixel corresponds to the average number of animals per km^2^ of suitable land in the census unit. The AW models were introduced because the spatial predictor variables used in the downscaling algorithms (e.g. human population density, vegetation indices and topography) may introduce uncontrolled confounding effects or circularity for users wishing to study livestock distribution numbers independently of any other spatial variables. The AW models are free of the influence of other spatial predictor variables, at the cost of displaying cruder distribution patterns, especially in large census areas containing a wide range of different environmental, land-use and farming conditions. In polygons where input census data were missing, the AW model simply includes the aggregated predictions of the DA models, and a separate layer is provided for the user that distinguishes between predictions and census observations.

GLW 3 provides global data (DA, AW and prediction status) at a spatial resolution of 0.083333 decimal degrees (approximately 10 km at the equator), as the higher spatial resolution of previous GLW versions could be misleading in areas where the census data were of poor quality.

Future versions of GLW will differentiate stocks according to production systems for ruminant (meat vs. dairy) and monogastric species (intensive vs. extensive, meat vs. egg production). Higher resolution models for individual countries where the census data can support such predictions will also be produced.

## Methods

The only change to the overall workflow, which was fully detailed for GLW 2 in Robinson *et al.*^8^, is that in GLW 3 RF models have replaced stratified linear multiple regressions for predictions^9^.

### Data mining

**Figure 1:**
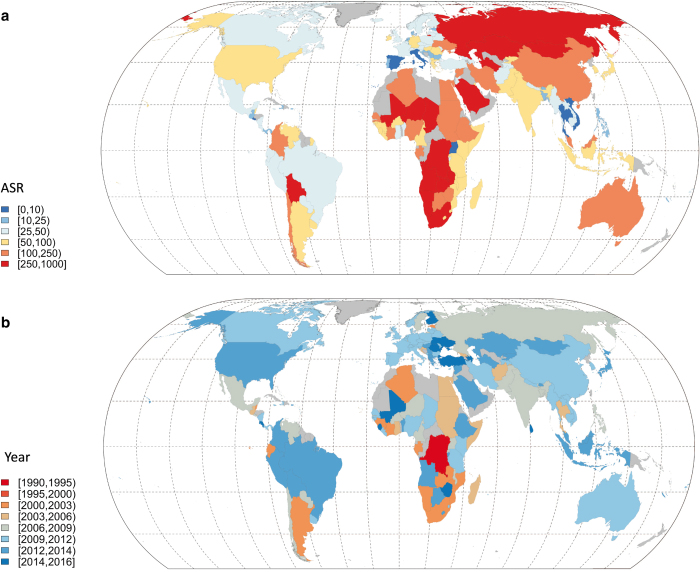
Maps of the GLW 3 average spatial resolution (ASR) and year of the chicken census data. Countries where no subnational census data could be obtained are indicated in grey.

**Figure 2:**
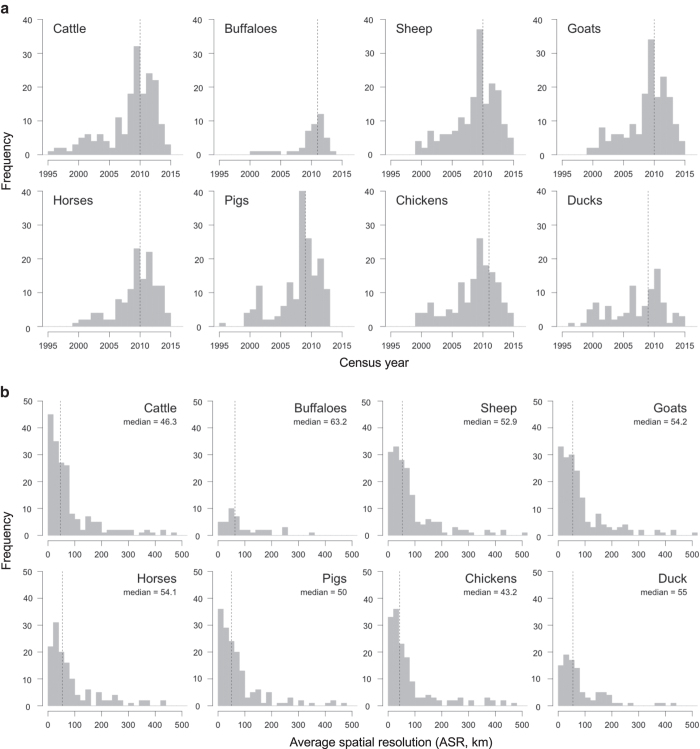
Distribution of input census data year and average spatial resolution per country for the different livestock species. The median year of the subnational census (**a**) is indicated with the vertical dotted line. The median spatial resolution (**b**) ranges between 43.2 and 55 km, and shows considerable variability, with some countries having an ASR well above 100 km (corresponding to 10,000 km^2^).

Detailed livestock census statistics are mined from agricultural yearbooks or through direct contacts with ministries or statistical bureaux. The census statistics are usually found in the form of numbers per administrative unit, in which case they need to be linked to corresponding geographic information system (GIS) boundaries. Data are increasingly found though as pre-prepared GIS files that are then integrated in a centralized database. These individual country data are combined into a global database, which often implies resolving typology issues; miss-matched, split or merged polygons, for example. In compiling GIS data from subnational census counts priority is given to censuses that most closely match the reference year (2010 for GLW 3) and those with the highest level of spatial detail. This results in a global mosaic of data from different spatial resolutions and different years. For example, Fig. 1a illustrates the heterogeneity in the Average Spatial Resolution (ASR) - the square root of the mean area of the census units - of the input data in each country for chickens. Figure 1b shows the year of each census. These figures highlight the large variability in ASR, with countries such as Italy and Thailand having very detailed subnational data (ASR<10 km and a mean area<100 km^2^), and countries such as Russia or South Africa with very coarse subnational census data (ASR > 250 km and mean area > 62 500 km^2^). There are also important differences in the census years. The oldest subnational census data are for the Democratic Republic of the Congo (1994), whilst some countries, for example Turkey, have very recent data (2014). Both the ASR and year of the census data depend on the species in question, as sometimes data can be available for one species and not for others. The two types of information are essential indicators of data quality and are therefore provided in the metadata. Summarized distributions of ASR and census years of GLW 3 are shown for all species in Fig. 2a and b, and are detailed in the metadata for each species.

### Estimating densities corrected for unsuitable areas

Densities are estimated in each of the census polygon by dividing the number of animals from the census by the surface area of the administrative unit polygon (estimated in an Albert equal area projection), corrected by a mask excluding unsuitable areas. The suitability mask is very conservative and only excludes permanent water (pixels covered by >50 percent of water, see data source in Table 1), and areas where human population densities exceed 5,000 (North America, Europe and Oceania), 7,500 (South America) or 10 000 (Asia and Africa) people km^-2^ as defined by the human population data layer (see data source in Table 1). Those different thresholds are used to account for the fact that urban population density is often higher in LMICs, where small-scale livestock farming may continue deeper into peri-urban and urban areas. The thresholds were conservatively defined to exclude only the core urban centres following an exploratory data analysis of human population density in urban pixels defined by the MODIS global land cover 2010^10^ or with >50% built up areas in the Global Human Settlement Layer of 2014^11^. In addition, a global mask of protected areas is derived from the 2010 version of the World Database on Protected Areas (Table 1). The International Union for the Conservation of Nature (IUCN) categories Ia and Ib, II, and III were masked as unsuitable as these are characterised by stringent conservation measures and tight regulation of human activity – the encroachment of roaming cattle and other grazing activities is therefore less likely in these than in other areas^12^.

### Sampling and extraction of predictor covariates

Sampling points are distributed across the geographical space and the values for the suitability-corrected livestock densities are extracted from the subnational census data, constituting the dependent variable. The values of the predictor variables, listed in Table 1, are extracted for each of the sample pixels. All GIS raster layers of inputs (e.g. masks and predictor variables) and outputs (DA and AW predicted densities) are processed with a global extent and a spatial resolution of 5 minutes of arc, i.e. 0.083333 decimal degrees, which corresponds approximately to 10 km at the equator. The sampling strategy is identical to that described in Robinson *et al.*^8^, i.e. balancing the sampling between the most detailed census data while ensuring a sufficient geographical coverage in areas with less detailed input data. As a result, a minimum of one sampling point is taken from each census polygon, and additional points are added proportionally to the polygon surface area with a sampling density of one point for every 10 000 km^2^.

### Random Forest models and cross-validation

The sample points are divided between training and validation sets according to the subnational census polygons, using sample points from 70 percent of the polygons for training the models and from 30 percent of the polygons for assessing the model accuracy. This operation is repeated 5 times, each time selecting a different set of polygons to train the RF models and goodness-of-fit (GOF) measurements. The parameters of the RF models were investigated in Nicolas *et al.*^9^ and are set as follows: i) a third of the variables are used to build each tree with a minimum of 5 variables; ii) the number of trees is set to 1/20^th^ of the number of sampling points, with a minimum of 100 trees; and iii) the node size is set to 1/1,000^th^ of the number of sampling points, with a minimum node size of 5. The 5 bootstrapped RF models are then applied to the raster predictor variables to estimate a density value in each pixel, and the 5 predicted values are used to estimate the prediction mean and standard deviation in each pixel.

The 30 percent of polygon data that were held back for each bootstrap are used to estimate GOF metrics. For each of these polygons and each bootstrap, the predicted values of the pixels falling into the polygon are summed to calculate a predicted total per polygon. The predicted and observed animal numbers of these 30% validation polygons are then used to estimate the Root Mean Squared Error (RMSE) and correlation coefficient between the observed and predicted totals, as measures, respectively, of accuracy and precision (see technical validation section).

The entire analysis is stratified by developing 5 bootstrapped models for each continent: North America, South America, Europe, Africa, Asia and Oceania. In total 30 RF models are produced and applied to six continents. The GOF metrics are produced separately for each continent.

### Post-processing

For the dasymetric product, the average values predicted by the RF models are used as weights to distribute the animals within each subnational census unit at the pixel level. For each polygon, the pixel weights are multiplied by the ratio of the total number of animals per census unit to the sum of pixel weights in the polygon (i.e. total of the RF model mean prediction). In polygons with missing census values the RF mean predicted density is used (i.e. the factor applied to the weights=1). In some countries, the spatial census units have smaller areas than the area of a single pixel (approx. 100 km^2^ at the equator). In these cases, the sum of the animals from those small census polygons falling within a pixel is estimated and assigned to that pixel; replacing the RF model prediction. This can result in two situations. When pixels are smaller than their corresponding polygon, the sum of the pixel values matches the observed animal total of the polygon. Conversely, when pixels are larger than their corresponding polygons, the pixel value matches the sum of the intersecting smaller polygons. In both situations, issues of polygon boundaries going across pixel boundaries are resolved according to the proportion of the intersecting surface area.

For the AW product, when pixels are smaller than the corresponding census polygons, all pixels are given an equal weight, and animals are distributed homogenously within the census polygon, excluding unsuitable areas, where the animal density is set to 0. In pixels that are larger than the polygon size, the total animals from the polygons falling into the pixel are summed. In polygons where there were missing data in the global merge, the sum of the RF model mean prediction is used as an estimate of the total number of animals in the polygon, and these are distributed homogeneously in the same way as the observed census numbers.

In both the DA and AW products, pixels falling within polygons with missing animal totals are marked in a separate layer, so that users can distinguish densities derived from observed census numbers from those predicted by the RF models.

Finally, all pixels are corrected by a country factor so that the summed values of the pixel match the total number of animals registered in the FAOSTAT database for the reference year 2010. This ensures that subnational census data from different years are standardized to 2010 and that all totals are compatible with the numbers officially declared by countries to FAO. However, the original subnational country census data are provided in the metadata table. Users may revert to the original totals by applying the inverse country-level correction factor if needed.

## Code availability

The code is fully operational under R 3.3.3^13^ and the key packages were raster 2.5–8^14^, rgdal 1.2–13^15^, maptools 0.9–2^16^ and randomForest^17^. The full code used to implement GLW is available from the authors with no restriction but is currently provided with no detailed documentation.

## Data Records

**Figure 3:**
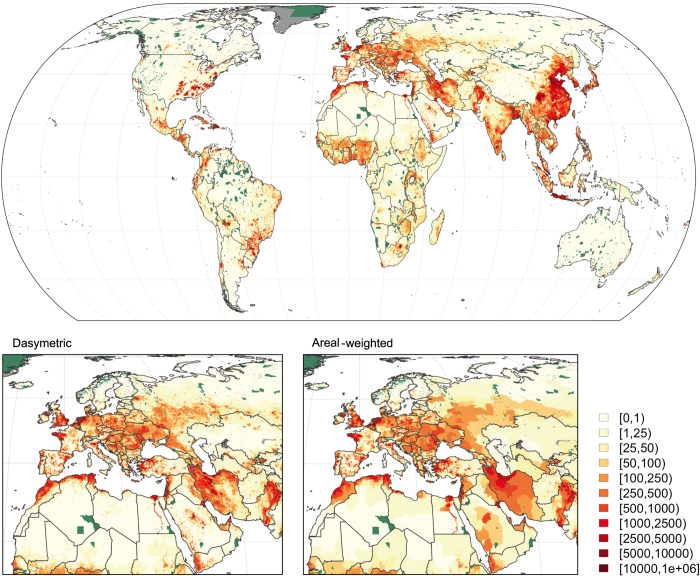
Distribution of chicken density in the world and within Europe. The bottom panels highlight the difference between the dasymetric (bottom left) and areal-weighted (bottom right) databases. Dark grey are areas considered unsuitable and dark green areas correspond to IUCN protected areas.

The data records described in this paper are publicly and freely available on the Gridded Livestock of World 3 Dataverse (Data Citation 1, 2, 3, 4, 5, 6, 7, 8) and through the FAO livestock systems World Web site (http://www.fao.org/livestock-systems/). The data records are grouped by species (Table 2), and each species data record includes a metadata document, quick view graphic files and the GIS data as Geotiff files with a spatial extent of −180 to 180 degrees of longitude and −90 to 90 degrees of latitude. With a spatial resolution of 0.083333 decimal degrees per pixel, the resulting raster is 4,320 by 2,160 pixels (Table 3). The metadata document provides a detailed explanation of the different files, quick views of the different maps, ASR and census year maps and histograms, indicators of the RF models’ GOF and a comprehensive list of original data sources grouped by countries and providing references to the publication and/or URL of the original country census data. Quick views and GIS raster files are provided for the dasymetric product, the areal-weighted product, and the distribution of prediction vs. observed status, highlighting areas where there were missing census data and where RF predictions were used (Table 3). As an example, Fig. 3 presents the predicted global distribution of chickens in the dasymetric product (top). The small inserts allow the difference between the DA (left) and AW (right) products to be observed. In countries were input census units are very small, such as Italy and Spain, the difference is hardly noticeable. In contrast, the AW product displays large areas with equal density in countries with large census units such as Russia and Iran, where the DA product redistributes chickens within census units according to the RF weights.

## Technical Validation

**Figure 4:**
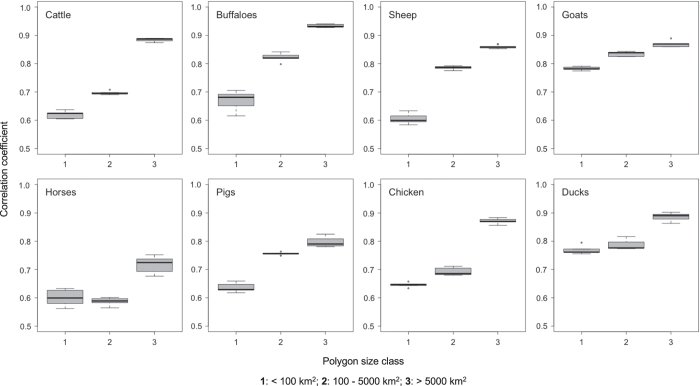
Correlation coefficients between observed and predicted livestock densities broken down by polygon census size and species.

The technical validation was carried out internally by training the models with 70 percent of the input polygon data and evaluating the predictions using 30 percent of the polygons that were not used to train the model. The GOF was evaluated using both the RMSE and the correlation coefficient between the observed and predicted log-transformed numbers of animals per polygon. However, in the event that the RMSE and correlation coefficient were similar, we only report the correlation coefficient as an indicator of GOF. Figure 4 shows the GOF plot broken down by species and polygon size class. Individual GOF plots broken down by polygon size class and continent are provided in the individual metadata files for each species.

The GOF was moderate to high, depending on species and size of census administrative unit (Fig. 4). Since our spatial model predicts values at a spatial resolution of roughly 10 km (at the equator), the GOF metric of the first polygon size class (<100km^2^) gives the most accurate estimate of the prediction accuracy of the pixel-level predictions. These ranged between 0.60 and 0.78, meaning that a significant part of the variability is not captured by the model and that pixel-level estimates cannot be assumed to fully represent what is on the ground. This could be linked to important predictor variables that are absent from the model, or to the stochastic nature of the spatial allocation of farms. As spatial units become larger, the variability gets filtered out and the observed values become easier to predict, with correlation coefficients close to 0.90 for the largest units. So, the gap-filling capacity of the models can be assumed to be good, which benefits both the DA and AW products. The GOF metrics by continent and species sometime reflect very different model qualities depending on the continent. Future studies should evaluate whether alternative stratifications could better harmonize the quality of the models across geographic regions. For example, groupings based on the economic status of countries may prove more appropriate than those based on continents. Livestock farming is constrained by production factors such as land, capital and manpower, and the last two are strongly associated with countries’ economic status, which influences how farms can be distributed across the landscape.

The GOF metrics need to be interpreted with care because they result from internal cross-validation. They do not measure the correspondence between the predicted densities of animals and what is actually on the ground. If the census itself is of poor quality, there could be discrepancies between the recorded numbers and what is actually on the ground, let alone between the census and the predicted values. Furthermore, census data are mostly based on where animals are registered to their owner, not necessarily where they are raised or spend most of their lifetime. For ruminants raised in pastoral systems, or ducks raised in free-grazing systems, for example, there could be significant seasonal changes in the spatial distributions of animals that would not be captured in the models.

The outputs also assume no livestock to occur in IUCN protected areas, and imposing a density of zero in such areas. It is, however, known that livestock encroach on protected areas and the validity of these assumptions depends on how effectively these restrictions are enforced. This varies greatly from country to country and even within countries.

One possible way to validate the models would be to use household demographic and health surveys (DHS) or living standards measurement studies (LSMS) data on livestock ownership. These data are typically geo-referenced at the cluster level, follow a completely different sampling approach and would therefore provide a different base for the development of livestock models. Cross-checking the results of models derived from large-scale census and from point-based surveys would help to identify areas of convergence where predictions would be consolidated, and areas of divergence where there would be higher uncertainties associated with the predictions. Field observations and aerial surveys provide efficient means of collecting high-quality data that could be used to validate the models, however, they are costly, especially when carried out over large areas. The cost-effectiveness of remote sensing for counting large animals was recently reviewed, and appears currently to be of limited utility^18^.

## Usage Notes

This version of GLW is suited to applications in the domains of socio-economics, environment and health. The data are most appropriate for applications at global and continental scales. Decisions regarding the use of this version of GLW over smaller spatial extents should be taken in relation to the ASR of the underlying census data. For example, analyses of GLW data in Brazil, Spain or Thailand would be appropriate because their respective ASRs are small relative to the size of the country. In contrast, we would discourage the country-level use of GLW data in countries such as Russia or Mali, because the ASR values are particularly high (>250 km). It is also important that users of these data are mindful of the fact that the type of production system is not accounted for in these livestock distribution data. The diverse contexts in which livestock are raised have major bearings on their primary uses, their productivity, the benefits they confer, the constraints to production and the impacts they have. The distribution data should therefore be used in conjunction with information on production systems. Currently, global^1^ and regional^19^ ruminant production systems data are available and global monogastric systems are available for pigs and chickens^20^.

The DA version is recommended for applications where spatial detail matters more than concerns about circularity in the analytical workflow in relation to spatial predictor covariates. However, we warn potential users against over-interpretation of spatial accuracy of the DA product. As indicated by the GOF metrics, much variability was not captured by the models and the downscaled densities of the DA version only imperfectly represent what may actually be on the ground. When circularity concerns are more important than detailed spatial resolution, it is recommended that the AW versions be used.

This is the third version of GLW, and the previous version had reference years of 2002 and 2006, respectively. However, since the three version of GLW differ in the type of input data, the predictor covariates and modelling methods, we would discourage their use for time-series analysis. Future studies will develop appropriate models to map how livestock distributions have changed over time.

In order to facilitate zonal summations, the values in each pixel of the DA and AW data sets correspond to the absolute numbers of animals, not to densities. These values can be converted to densities (number per km^2^) by dividing each pixel value by the pixel area in km^2^. For convenience, a global Geotiff file of pixel areas, expressed in km^2^/pixel, is provided along with each species data file (Table 3).

**Figure 5:**
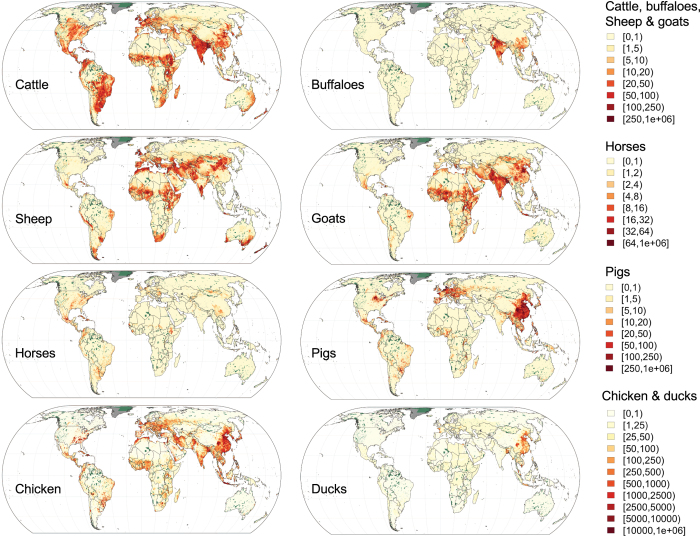
Overview of the Gridded Livestock of the World (GLW 3) data sets for cattle, buffaloes, sheep, goats, horses, pigs, chickens and ducks, based on the dasymetric model. Dark grey are areas considered unsuitable and dark green areas correspond to IUCN protected areas.

All outputs have been corrected so that the total number of animals in a country matches the FAOSTAT 2010 total stock. However, in a number of cases, there are significant differences between the total numbers of animals found in the original national census data and the values recorded in FAOSTAT. The total of the original census is provided for each country and species in the metadata report so that users may revert to these by dividing all pixel values by the FAOSTAT 2010/Census total ratio. Figure 5 shows the global distributions of the eight livestock species included based on the DA models.

## Additional information

**How to cite this article**: Gilbert. M. *et al.* Global distribution data for cattle, buffaloes, horses, sheep, goats, pigs, chickens and ducks in 2010. *Sci. Data*. 5:180227 doi: 10.1038/sdata.2018.227 (2018).

**Publisher’s note**: Springer Nature remains neutral with regard to jurisdictional claims in published maps and institutional affiliations.

## Supplementary Material



## Figures and Tables

**Table 1:** List of input spatial dataset used in the production of the GLW datasets.

Type	Variables	Use	Source
Land	Land and water area	Spatial domain	CIESIN^21^
Land use	IUCN world database of protected area	Mask	UNEP-WCMC^22^
Anthropogenic	Human population density (consensus model between Worldpop, Landscan and GPW4)	Spatial predictor and suitability mask	Tatem *et al.*^23^, Dobson *et al.*^24^, CIESIN^25^
	Travel time to cities of 50,000 people	Spatial predictor	Nelson *et al.*^26^
Topography	Elevation (GTOPO30)	Spatial predictor	LDAAC^27^
	Slope (GTOPO30)	Spatial predictor	LDAAC^27^
Vegetation	10 Fourier-derived variables from Normalized Difference Vegetation Index from MODIS (MODIS)^∗^	Spatial predictor	Scharlemann *et al.*^28^
	Length of growing period	Spatial predictor	Jones & Thornton^29^
	Green-up and senescence (annual cycle 1 and 2)	Spatial predictor	Zhang *et al.*^30^
	Cropping intensity	Spatial predictor	Fritz *et al.*^31^
	Forest cover	Spatial predictor	Hansen *et al.*^32^
Climatic	14 Fourier-derived variables from Day Land Surface Temperature (MODIS)	Spatial predictor	Scharlemann *et al.*^28^
	Precipitations	Spatial predictor	Hijmans *et al.*^33^
^∗^Annual mean, annual muinimum, annual maximum, amplitude and phase of annual cycle, amplitude and phase of bi-annual cycle, amplitude and phase of tri-annual cycle, variance in annual, bi-annual, and tri-annual cycles.			

**Table 2:** List of data records, organised by species.

Species	ASR median	Year min./median/max.	Spatial units	Density Median/99% percentile	Data citation
Cattle	46.3 km	1984/2010/2014	74,035	12.98/277.42	1
Buffaloes	63.2 km	2002/2011/2013	31,900	0.0277/74.96	2
Sheep	52.9 km	1990/2010/2014	58,869	2.207/247.70	3
Goats	54.2 km	2000/2010/2014	51,039	0.695/418.30	4
Horses	54.1	1993/2010/2014	40,712	0.459/16.91	5
Pigs	43.3	2000/2010/2014	55,462	3.742/928.22	6
Chickens	43.2	1994/2010/2014	69,761	67.57/10,896	7
Ducks	54.4	1994/2009/2014	44,109	4.263/2,564	8

**Table 3:** List of files provided for each species, with chickens as an example.

File name	Description	File type
1_Ch_2010_Metadata.html	Full metadata document report	Hypertext Markup Language
2_Ch_2010_Da.png	Quick view graphic file of the dasymetric product	Portable Network Graphics
3_Ch_2010_Aw.png	Quick view graphic file of the areal-weighted product	Portable Network Graphics
4_Ch_2010_Ps.png	Quick view graphic file of the prediction status of the areal-weighted product	Portable Network Graphics
5_Ch_2010_Da.tif	GIS file of the dasymetric product, (absolute number of animals per pixel; 4320 by 2160 pixels of 0.083333 decimal degrees resolution)	Geotiff
6_Ch_2010_Aw.tif	GIS file of the areal-weighted product, (absolute number of animals per pixel; 4320 by 2160 pixels of 0.083333 decimal degrees resolution)	Geotiff
7_Ch_2010_Ps.tif	GIS file of the areal-weighted prediction status (1: from predictions, 0: from observed census; 4320 by 2160 pixels of 0.083333 decimal degrees resolution)	Geotiff
8_Areakm.tif	GIS file of the area per pixel (square km; 4320 by 2160 pixels of 0.083333 decimal degrees resolution)	Geotiff
For other species, the “Ch” prefix is replaced by “Ct” for cattle, “Bf” for buffaloes, “Sh” for sheep, “Gt” for goats, “Ho” for horses, “Pg” for pigs and “Dk” for ducks.		
